# Supplementary material for: Breakthrough Tick-Borne Encephalitis and Epidemiological Trends in an Endemic Region in Poland: A Retrospective Hospital-Based Study, 1988–2020
Source: Vaccines (Basel). 2025 Jun 20;13(7):665. doi: 10.3390/vaccines13070665 (PMC12300996; doi:10.3390/vaccines13070665)
Supplement: Supplementary file 1 [file vaccines-13-00665-s001.zip › vaccines-3665389-supplementary.pdf]

Table S1. Estimated cumulative uptake of completed TBE vaccine series and resulting population coverage in northeastern Poland (Podlaskie Voivodship), 1999–2020. Population estimates are based on regional census data; coverage (%) reflects the proportion of the population with completed primary vaccination (3 doses) according to National Institute of Public Health data. Booster dose adherence is unknown.

Year	Population	Annual number of vaccinated	Cumulative number of vaccinated	Coverage (%)
1999	1212269	2385	2385	0.196738513
2000	1210688	2308	4693	0.387630835
2001	1209439	2467	7160	0.592010015
2002	1208606	3373	10533	0.871499893
2003	1205117	10165	20698	1.717509586
2004	1202425	2540	23238	1.932594549
2005	1199689	2780	26018	2.168728729
2006	1196101	3080	29098	2.432737704
2007	1192660	3665	32763	2.747052806
2008	1191470	3287	36050	3.025674167
2009	1189731	4460	40510	3.404971376
2010	1203448	3574	44084	3.663141241
2011	1200982	3142	47226	3.932282082
2012	1198690	2402	49628	4.14018637
2013	1196485	2642	52270	4.368629778
2014	1193348	2828	55098	4.617094092
2015	1188800	2397	57495	4.836389637
2016	1186625	3068	60563	5.103802802
2017	1184548	3528	64091	5.410586992
2018	1181533	4293	68384	5.787735087
2019	1178353	5541	73925	6.273586947
2020	1156591	3957	77882	6.733754629
1999–2020 mean	1194891	3540	77882	6.517917689

Note: Cumulative vaccine coverage increased gradually over the study period, reaching 6.73% by 2020. These data were used to estimate average population-level vaccine uptake in the screening-method calculation of vaccine effectiveness (VE). The mean coverage across the period 1999–2020 was 6.52%.

Table S2. A detailed description of clinical course of tick-borne encephalitis in patients previously vaccinated with the TBE vaccine

**Patient 1**	A 41-year-old male, fully vaccinated but not boosted, from rural setting, hospitalized in 2002 with meningitis. He stayed 14 days, reporting muscle pain and headache with neck stiffness, positive Kernig's and Brudziński's signs, bradycardia, but no dizziness, nausea, or vomiting. Initial CSF cytosis was 65 cells/µl with lymphocyte predominance. No follow-up CSF analysis was reported.
**Patient 2**	A 25-year-old male from urban setting, incompletely vaccinated, hospitalized in 2002 with encephalomeningitis for 17 days. Symptoms included headache, dizziness, nausea, severe neck stiffness, positive Kernig's, Brudziński's, bilateral Oppenheim's signs, pyramidal signs, and bradycardia. Initial CSF cytosis was 60 cells/µl with 55% lymphocytes and protein of 37.8 mg/dl. No follow-up CSF analysis was reported.
**Patient 3**	A 39-year-old male from a village, fully vaccinated and protected, hospitalized in 2008 with encephalomeningitis for 15 days. Symptoms were headache, dizziness, vomiting, and cerebellar symptoms. Initial CSF cytosis was 87 cells/µl with 77% lymphocytes and protein of 124 mg/dl. Subsequent CSF analyses showed cytosis of 49 (day 19) and 30 (day 28), reflecting a gradual normalization.
**Patient 4**	A 38-year-old male from an urban background, incompletely vaccinated, hospitalized in 2008 for 12 days with meningitis. He experienced headache, dizziness, vomiting, and neck stiffness. Initial CSF cytosis was 140 cells/µl with 46% lymphocytes; follow-up on day 12 showed cytosis reduced to 34 cells/µl.
**Patient 5**	A 25-year-old female from a rural environment, incompletely vaccinated, hospitalized in 2008 with meningitis for 26 days. Symptoms included headache without meningeal signs, but with bilateral Oppenheim's signs and pyramidal signs. Initial CSF cytosis was 213 cells/µl with 92% lymphocytes, decreasing to 25 cells/µl by day 23.
**Patient 6**	A 47-year-old male from a rural setting, incompletely vaccinated, hospitalized in 2009 with encephalomeningitis for 13 days. He had headache, dizziness, vomiting, neck stiffness, decreased level of consciousness, and bradycardia. Initial CSF cytosis was 120 cells/µl with 58% lymphocytes. No follow-up CSF data available.
**Patient 7**	A 42-year-old female from an urban setting, fully vaccinated but not boosted, hospitalized in 2009 with TBE-like meningitic syndrome for 7 days. Symptoms were muscle pain and headache. CSF cytosis was minimal at 2 cells/µl.
**Patient 8**	A 63-year-old female from an urban area, incompletely vaccinated, hospitalized in 2011 with meningitis for 15 days. Symptoms included headache, vomiting, Kernig's sign, bilateral Oppenheim's signs, and pyramidal signs. Initial CSF cytosis was 28 cells/µl with 64% lymphocytes. No follow-up CSF data available.
**Patient 9**	A 26-year-old male from a rural setting, fully vaccinated and protected, hospitalized in 2012 with encephalomeningitis for 24 days. Symptoms included headache, vomiting, Kernig's sign, bilateral Oppenheim's sign, pyramidal signs, tremor, speech disturbance, decreased level of consciousness. Initial CSF cytosis was 358 cells/µl with 86% lymphocytes and protein of 95.4 mg/dl. Follow-up CSF analyses showed cytosis of 45 (day 13), 42 (day 19), and 18 (day 56).
**Patient 10**	A 43-year-old female from an urban area, incompletely vaccinated, hospitalized in 2015 with meningitis for 14 days. Symptoms included muscle pain, headache, dizziness, and cerebellar symptoms. Initial CSF cytosis was 144 cells/µl with 43% lymphocytes, decreasing to 115 cells/µl by day 13.
**Patient 11**	A 45-year-old male from an urban setting, incompletely vaccinated, hospitalized in 2018 with meningitis for 12 days. Symptoms included headache, dizziness, nausea, and cerebellar symptoms, but no meningeal signs. Initial CSF cytosis was 365 cells/µl with 93% lymphocytes and protein of 107 mg/dl. No follow-up CSF data available.

**Patient 12**	A 63-year-old female, incompletely vaccinated, hospitalized in 2019 with meningitis. She reported headache, and nausea. Initial CSF cytosis was 41 cells/µl with 80% lymphocytes. No follow-up CSF data available.
**Patient 13**	A 53-year-old female from a rural setting, incompletely vaccinated, hospitalized in 2020 with meningoencephalomyelitis. Symptoms included headache, dizziness, nausea, tremor, sensory disturbances related to cranial nerves, and cerebellar symptoms. Initial CSF cytosis was 96 cells/µl with 94% lymphocytes. Follow-up CSF revealed 43 cells/µl.

Table S3. Cerebrospinal fluid analysis in patients diagnosed with tick-borne encephalitis who were previously vaccinated with the TBE vaccine.

PATIENT	SEX	AGE	DIAGNOSIS	VACCINATION STATUS	INITIAL CSF RESULTS	FOLLOW-UP CSF RESULTS
**1**	M	41	Meningitis	Fully vaccinated, not boosted	Cytosis 65<br>Lymphocytes 98%<br>Protein 65.5	None
**2**	M	25	Encephalomeningitis	Incompletely vaccinated	Cytosis 60<br>Lymphocytes 55%<br>Protein 37.8	None
**3**	M	39	Encephalomeningitis	Fully vaccinated	Cytosis 87<br>Lymphocytes 77%<br>Protein 124	Cytosis 49 (day 19), 30 (day 28)
**4**	M	38	Meningitis	Incompletely vaccinated	Cytosis 140<br>Lymphocytes 46%<br>Protein 73.4	Cytosis 34 (day 12)
**5**	F	25	Meningitis	Incompletely vaccinated	Cytosis 213<br>Lymphocytes 88%<br>Protein 73.8	Cytosis 25 (day 23)
**6**	M	47	Encephalomeningitis	Incompletely vaccinated	Cytosis 120<br>Lymphocytes 58%<br>Protein 104.4	None
**7**	F	42	Meningitic syndrome	Fully vaccinated, not boosted	Cytosis 2<br>Lymphocytes unk<br>Protein 22.7	None
**8**	F	63	Meningitis	Incompletely vaccinated	Cytosis 28<br>Lymphocytes 64%<br>Protein 35.5	None
**9**	M	26	Encephalomeningitis	Fully vaccinated	Cytosis 358<br>Lymphocytes 86%<br>Protein 95.4	Cytosis 45 (day 13), 42 (day 19), 18 (day 56)
**10**	F	43	Meningitis	Incompletely vaccinated	Cytosis 144<br>Lymphocytes 43%<br>Protein 47	Cytosis 115 (day 13)
**11**	M	45	Meningitis	Incompletely vaccinated	Cytosis 365<br>Lymphocytes 93%<br>Protein 107	None
**12**	F	63	Meningitis	Incompletely vaccinated	Cytosis 41<br>Lymphocytes 80%<br>Protein 39	None
**13**	F	53	Meningoencephalomyelitis	Incompletely vaccinated	Cytosis 96<br>Lymphocytes 94%<br>Protein 64	Cytosis 43

Cytosis is presented as cells/μL, protein as mg/dL; age is presented in years.
